# Cognitive and Academic Outcomes of Fundamental Motor Skill and Physical Activity Interventions Designed for Children with Special Educational Needs: A Systematic Review

**DOI:** 10.3390/brainsci12081001

**Published:** 2022-07-28

**Authors:** Pinja Jylänki, Theo Mbay, Anni Byman, Airi Hakkarainen, Arja Sääkslahti, Pirjo Aunio

**Affiliations:** 1 Faculty of Educational Sciences, University of Helsinki, 00170 Helsinki, Finland; theo.mbay@helsinki.fi (T.M.); anni.byman@helsinki.fi (A.B.); airi.hakkarainen@helsinki.fi (A.H.); pirjo.aunio@helsinki.fi (P.A.); 2 Faculty of Sport and Health Sciences, University of Jyväskylä, 40600 Jyväskylä, Finland; arja.saakslahti@jyu.fi

**Keywords:** academic skills, cognition, early intervention, motor skills, physical activity, special educational needs, systematic review

## Abstract

This systematic review aimed to investigate the methodological quality and the effects of fundamental motor skills and physical activity interventions on cognitive and academic skills in 3- to 7-year-old children with special educational needs. The review was reported in accordance with the Preferred Reporting Items for Systematic Reviews and Meta-Analyses (PRISMA 2020) statement. A literature search was carried out in April 2020 (updated in January 2022) using seven electronic databases, including ERIC, Scopus, Web of Science, PsycINFO, CINAHL, PubMed, and SPORTDiscus. The methodological quality of the studies was assessed with Effective Public Health Practice Project (EPHPP) Quality Assessment Tool. Cohen’s *d* effect sizes and post-hoc power analyses were conducted for the included studies. Altogether 22 studies (1883 children) met the inclusion criteria, representing children at-risk for learning difficulties, due to family background (*n*_studies_ = 8), children with learning difficulties (*n*_studies_ = 7), learning disabilities (*n*_studies_ = 5), and physical disabilities (*n*_studies_ = 2). Two of the included 22 studies displayed strong, one moderate, and 19 studies weak methodological quality. The intervention effects appeared to be somewhat dependent on the severity of the learning difficulty; in cognitive and language skills, the effects were largest in children at-risk due to family background, whereas in executive functions the effects were largest in children with learning disabilities. However, due to the vast heterogeneity of the included studies, and a rather low methodological quality, it is challenging to summarize the findings in a generalizable manner. Thus, additional high-quality research is required to determine the effectiveness of the interventions.

## 1. Introduction

Children’s cognitive (e.g., executive functions) and academic skills (e.g., early numeracy and literacy skills) start to develop during the early years [1,2] which provide important grounds for later development [3]. During these years, in particular, the development of cognitive and academic skills is highly interrelated [4]. Thus, early childhood education has an important role in children’s development, especially for children with special educational needs (SEN) [3] whose later academic success is at risk [5]. Children with SEN are not a homogeneous group, but rather include a wide range of children with various types and extents of learning difficulties or disabilities [4,6]; stemming, for instance, from biological, neurobiological, intellectual, genetic, or environmental factors [7]. While the challenges of children with SEN differ widely, in general, a requirement for customized special education is observed [4]. Early childhood education provides a valuable environment for the implementation of effective interventions to support the learning of children with SEN, and thereby minimize or prevent the impact of learning difficulties, disabilities, or at-risk conditions on children’s development and future capabilities [4,8].

Fundamental motor skills (FMS, i.e., balance, and manipulative and locomotor movement skills) [9] and physical activity (PA; i.e., bodily movements that increase energy expenditure) [10] have been found to be related to cognitive and academic skills in children [11,12]. The close relationship between FMS and cognitive skills has been explained by co-activation in the same brain areas (i.e., cerebellum, prefrontal cortex, and basal ganglia) [13]. In addition, studies have shown that the relationship between PA and cognitive skills may be mediated through improved executive functions, especially inhibition [12,14], and that particularly high-intensity PA may affect cognitive skills through changes in the brain, via increased cerebral blood volume, and other physiological changes, such as increased neurogenesis [11,14,15].

In recent decades, studies examining the effects of FMS and PA interventions on cognitive skills have increased rapidly [12]. A recent systematic review demonstrated the positive effects of FMS and PA interventions on preschoolers’ cognitive and academic skills in typically developing children [16]. However, the effects, as well as the quality of FMS and PA interventions on children with SEN have not been previously analyzed. Considering that children with SEN are at risk for developing more severe problems in their academic skills during later years [17], it is highly important to investigate the most effective evidence-based practices for supporting cognitive and academic learning at an early age [18]. As FMS and PA are associated with cognitive and academic skills already in early childhood [13], and FMS and PA interventions have been found to improve typically developing children’s cognitive and academic skills [16], it is plausible that FMS and PA interventions similarly support the learning of children with SEN. Thus, the aim of the present systematic review was to investigate the methodological quality and the effects of FMS and PA interventions on cognitive and academic skills in children aged 3–7 years-old with SEN. Since children with SEN include a wide range of children with various types and extents of learning difficulties or disabilities [4,6], the intervention effects were examined in groups based on the assumed severity of the children’s learning difficulties.

## 2. Materials and Methods

The present systematic review was reported in accordance with the Preferred Reporting Items for Systematic Reviews and Meta-Analyses (PRISMA 2020) statement, which is designed for systematic reviews that evaluate the effects of intervention studies [19]. The review protocol was not pre-registered. The systematic review was conducted as follows:

*Step 1*: A literature search, including abstract rating and full-text screening, based on the pre-determined eligibility criteria, was carried out in April 2020 by two authors (P.J. and T.M.).

*Step 2*: In January 2022, an updated literature search was carried out for studies published between April 2020 and January 2022 by two authors (T.M. and A.B.) following the aforementioned protocol.

*Step 3*: Methodological quality of the studies was assessed with the Effective Public Health Practice Project Quality Assessment Tool for Quantitative Studies (EPHPP).

*Step 4*: Cohen’s *d* effect sizes were calculated, and post hoc power analyses were conducted to determine the statistical power of the included studies.

### 2.1. Eligibility Criteria

Peer-reviewed intervention studies investigating the effects of FMS or PA interventions on cognitive or academic skills in preschoolers with SEN published in English, were eligible for the present systematic review. Specific eligibility criteria are reported according to the PICO framework [20]:

*Population*: Published peer-reviewed intervention studies that were published in English and included children aged 3–7-years old with SEN. For instance, children at risk for learning difficulties due to family background (e.g., low socioeconomic status (SES)), children with learning difficulties (e.g., high risk of attention deficit hyperactivity disorder (ADHD)), learning disabilities (e.g., autism spectrum disorder), and physical disabilities (e.g., cerebral palsy) were included.

*Intervention*: All intervention studies with only FMS and/or PA practices or a program that combined FMS and/or PA with cognitive or academic skill practices (e.g., children counting balls while playing with them or collecting items in a particular order) [21] were included. For study designs, all intervention designs except case studies were included. Considering that the present study focused on children with a wide range of SEN (e.g., children with cerebral palsy) it may prove difficult to find a comparable control group, and thus, this was not required.

*Comparator*: The business-as-usual control groups were used in the analysis as a comparator. Studies that used an active control group were analyzed as separate interventions.

*Outcome*: The effects of the intervention had to be assessed via cognitive or academic outcomes, such as measures of cognitive, language, and numeracy skills or executive functions.

The systematic literature search was carried out on 16 April 2020, by two authors, P.J. and T.M., using seven electronic databases, including ERIC, Scopus, Web of Science, PsycINFO, CINAHL, PubMed, and SPORTDiscus. Since there was more than one year from the previous literature search, an updated search, following the same methods, was carried out on 18 January 2022 by the authors T.M. and A.B. Search terms were designed in accordance with the PICO framework [20] and consisted of the following: “early education” OR child * AND motor * OR “physical activity” AND intervention OR program * OR treatment OR training OR instruction AND cognit * OR academic *. When possible, additional search filters were used to exclude studies that investigated children older than seven years, and in the updated literature search only articles published from 2020 onwards were examined.

### 2.2. Study Selection

Three authors performed the article selection according to the predetermined eligibility criteria. The articles were initially screened based on the abstract. In terms of inclusion, the abstracts were coded as “yes”, “maybe” or “no”. The inter-rater agreement was determined during the abstract rating processes by calculating Cohen’s weighted kappa. In the first literature search, both authors rated the first 40% (*n* = 2266) of the abstracts independently, after which the inter-rater agreement was 0.718 and the remaining abstracts were divided between the authors. In the updated literature search, both authors rated all of the abstracts (*n* = 2198) independently with an inter-rater agreement of 0.771. In both cases, the inter-rater agreement could be considered as good [22]. Following the abstract screening, all of the eligible articles underwent full-text screening, where the authors independently decided whether to “exclude”, “include” or “maybe” include each article.

### 2.3. Methodological Quality

The methodological quality of the eligible studies was assessed with the Effective Public Health Practice Project Quality Assessment Tool for Quantitative Studies (EPHPP) [23,24]. The tool is suitable for evaluating the quality of a variety of study designs (e.g., randomized controlled trials (RCT) and pre-post designs (PPD)) [25] and has been used previously in systematic reviews in this particular field [16,26]. The inter-rater agreement has been shown to be more consistent with the EPHPP tool compared to the Cochrane Collaboration Risk of Bias Tool [25], for instance. Three authors rated each study procedure as “strong”, “moderate” or “weak”. Final ratings were formed based on six sections (selection bias, study design, confounders, blinding, data collection methods, and withdrawals and drop-outs) with the following criteria: studies with no weak ratings and at least four strong ratings were considered as “strong”; studies with less than four strong ratings and one weak rating were considered as “moderate”; studies with two or more weak ratings were considered as “weak”. For more detailed criteria see the Quality Assessment Tool for Quantitative Studies Dictionary [23]. Any disagreements were solved in consensus meetings with all authors.

### 2.4. Data Extraction

Data were extracted from the eligible studies independently by three authors. Extracted data included the geographical location, study design, sample size, children’s age, gender, and reason for SEN, cognitive and academic outcomes, intervention exposure, intervention details (only FMS and/or PA interventions and combined interventions), control conditions, and data for effect size calculations. If missing data was encountered, the corresponding author was contacted in order to receive the required information.

### 2.5. Effect Size Calculations

Cohen’s d effect sizes [27] were calculated to allow for the quantification and comparison of the effects across the studies. Effect sizes were calculated for the studies that demonstrated significant effects and provided sufficient information (i.e., pre- and post-scores, as well as the associated standard deviations or standard errors). If a study demonstrated significant effects for multiple outcomes, all of them were included. Cohen’s *d* effect sizes of <0.2, 0.2, 0.5, and 0.8, correspond to trivial, small, medium, and large effects, respectively [27].

Between-group effects were calculated in accordance with the following;

$$ES_{\left(d\right)}=\frac{\left(M_{post,\;E}-M_{pre,\;E}\right)-\left(M_{post,\;C}-M_{pre,\;C}\right)}{SDpooled_{pre}}\;$$

where

$$SDpooled_{pre}=\sqrt{\frac{\left(n_{E}-1\right)\;SD_{pre2,\;E}+\left(n_{C}-1\right)\;SD_{pre2,\;C\;})}{\left(n_{E}+n_{C}-2\right)}}$$

And within-group effects were calculated as:

$$ES_{\left(d\right)}=\frac{\left(M_{post}-M_{pre}\right)}{SD_{pre}}$$

*ES_(d)_* = Cohen’s *d* effect size
*M_post_* = mean post-score
*M_pre_* = mean pre-score
*E* = experimental group
*C* = control group
*SD_pooled_* = pooled standard deviation
*n* = sample size

### 2.6. Power Analyses

Power calculations were carried out with G*power 3.1.9.6 [28]. If a study had multiple groups, the power calculations were conducted on a sub-group basis in order to determine the power of specific group comparisons. Type 1 error probability (α) was computed as 0.05, corresponding to a significance level of 5%. A medium effect size (0.5) was used as the reference point to establish observed power for each outcome and a type 2 error probability (β) of 0.2, corresponding to a power of 0.8 (1 − β), or 80%, was selected as the cut-off point for adequate power [29].

## 3. Results

### 3.1. Search Results

**Figure 1:**
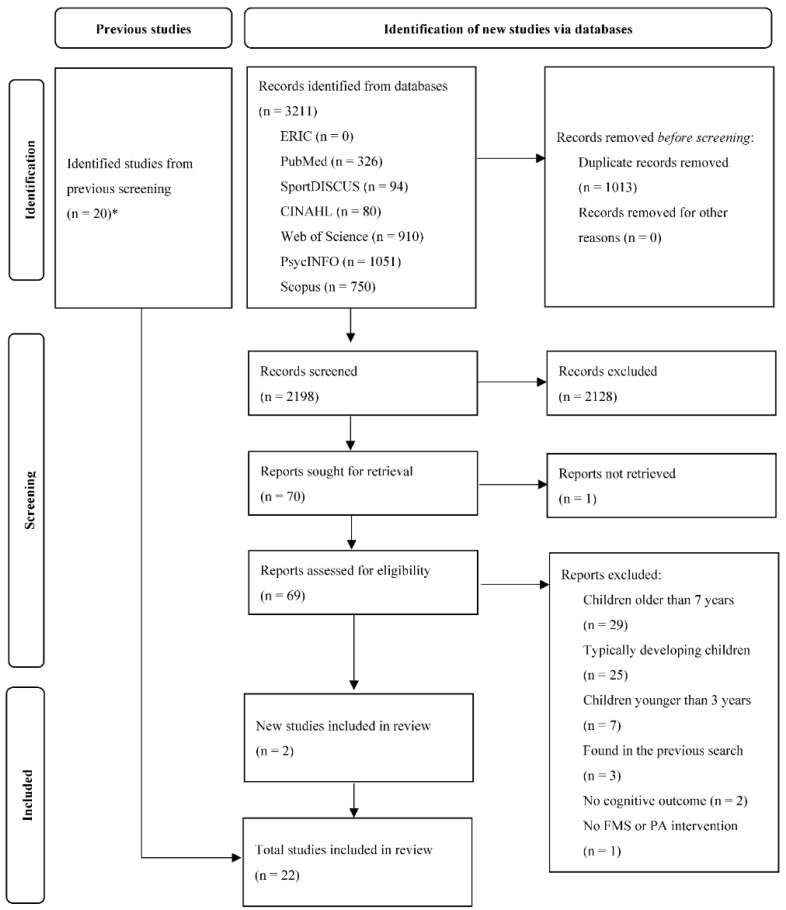
PRISMA flow diagram of the stages associated with the systematic selection of studies. * Studies were identified in the previous systematic review [16], but excluded since the review focused on typically developing children.

The stages of the systematic selection of the studies are presented in detail in Figure 1. In the updated literature search, a total of 3211 articles were found, which became 2198 articles after removal of duplicates. Of these, 2128 articles were excluded due to not meeting the eligibility criteria and the remaining 70 articles underwent full-text screening. Finally, 2 and 20 articles from the updated and the previous literature search (i.e., studies which were identified in the previous systematic review [16] but excluded since the review focused on typically developing children), respectively, were included.

### 3.2. Study Characteristics and Population

Study characteristics are presented in detail in Supplementary Table S1. The included 22 studies represented 1883 children with various types and extents of SEN. In order to compare the intervention effects, children were divided into four groups based on the assumed severity of the learning difficulty. Thereafter, the following groups were formed: children at-risk for learning difficulties due to family background (*n*_studies_ = 8; e.g., low SES) [30], children with learning difficulties (*n*_studies_ = 7; e.g., high risk of ADHD) [31], learning disabilities (*n*_studies_ = 5; e.g., autism spectrum disorder) [32], physical disabilities (*n*_studies_ = 2; e.g., cerebral palsy) [33]. The mean ages of the participants ranged from 3.8 [34] to 7.4 years [35], and all of the studies included both boys and girls, apart from two studies that only included the former [36,37]. In terms of geographical location, the included studies were conducted in ten countries representing North America, Europe, Asia, and Africa, and were published between 1972 [38] and 2021 [35].

### 3.3. Intervention Characteristics

In total, 22 studies with 25 intervention programs were included in the present review. Three of the studies [38,39,40] included two intervention programs that met the eligibility criteria, and thus, were analyzed separately. A total of 14 intervention programs focused on FMS only interventions [30,31,38,39,41,42,43,44], PA only interventions [35,40], or FMS and PA only interventions [32,45], while 11 programs combined FMS [3,21,33,36,37,46,47], PA [34,48], or FMS and PA [40,49] with cognitive or academic skill practices. Intervention duration ranged from five weeks [35] to one academic year [3,36,37,38,41,45]. The duration of sessions ranged from 10 min [43] to two and a half hours [49], and sessions were held once a week [21,42,46] to two times every preschool day [34,48]. Outcome measures were divided into five categories based on the provided descriptions: cognitive skills (e.g., Miller assessment for preschoolers) [36,37], executive functions (e.g., Childhood executive functioning inventory) [32], academic skills (e.g., Comprehensive test of basic skills) [39], language skills (e.g., Assessment of children’s language comprehension) [49], and numeracy (e.g., Counting and number recognition) [43].

### 3.4. Methodological Quality

The methodological quality was determined based on the following factors: study design, selection bias, confounders, blinding, data collection methods, and withdrawals and drop-outs [23]. Only two of the included 22 studies (9%) demonstrated strong methodological quality, while one study (5%) had moderate quality, and 19 studies (86%) were considered methodologically weak. The rating for each section, as well as the overall quality, of the studies is presented in Table 1.

Of the 22 included studies, 15 were controlled clinical trials (CCTs, i.e., quasi-experimental designs and RCTs that did not report the randomization process), three studies were RCTs, and the remaining four studies were PPDs. While only one study [30] referred the participants through randomization, in eight studies [3,36,37,38,39,41,43], the participants were referred from a source (e.g., preschool) in a systematic manner, and, thus, the participants were considered only somewhat likely to be representative of the target population. Important confounders (i.e., participants’ age, gender, health status, or pre-intervention score) were observed in four studies [21,36,37,40]. Of these, the confounders were 80–100% controlled in three studies [21,36,40], while in seven studies [3,32,34,41,43,45,48], no important confounders were observed between the groups. While most of the studies [3,21,30,33,34,37,38,39,41,42,44,46,47,48,49] did not report the outcome assessors’ blinding, six of the studies [31,35,36,40,43,45] reported that the outcome assessors were not aware, and only one study [32] reported that the outcome assessors were aware of the intervention or exposure status of the participants. Data collection methods were shown to be valid in seven studies [30,32,33,39,40,44,45] and of these, only three studies [30,40,44] reported that the data collection methods demonstrated good reliability in that particular data. Thus, only three studies [30,40,44] demonstrated strong quality in terms of data collection methods. Only five of the studies [21,32,44,45,48] reported both withdrawals and drop-outs in terms of numbers and reasons.

Of the included studies, ten (45%) were found to be underpowered to detect a medium effect size [31,34,36,38,39,40,43,44,46,48], while seven (32%) were confirmed to be adequately powered [3,21,30,32,35,41,45]; for the remaining five (23%) post hoc power could not be determined (i.e., within-group designs without required information) [33,37,42,47,49]. Only three (14%) of the included studies reported the conducting of a priori power analysis [32,36,37], and six studies stated small sample size as a limitation of the study [31,34,35,42,43,48]. It should be noted, that while underpowered to detect a medium effect size, in one study [36], the authors conducted a priori power calculations with a large (0.74) estimated effect size, based on a pilot study, for which the study was adequately powered.

**Table 1:** Methodological quality of the included studies.

Authors and Year	Selection Bias	Study Design	Confoun-ders	Blinding	Data Collection Methods	Withdrawals and Drop-Outs	Overall Quality Scores
Bala et al., 2013 [41]	moderate	strong	strong	moderate	weak	weak	weak
Berrol, 1984 [39]	moderate	strong	weak	moderate	moderate	weak	weak
Chevalier et al., 2017 [31]	moderate	strong	weak	moderate	weak	weak	weak
Coleman & Andersson, 1978 [49]	weak	moderate	weak	moderate	weak	weak	weak
Connor-Kuntz & Dummer, 1996 [40]	moderate	strong	strong	moderate	strong	weak	moderate
Devesa et al., 2011 [33]	weak	moderate	NA	moderate	moderate	weak	weak
Draper et al., 2012 [21]	weak	strong	weak	moderate	weak	moderate	weak
Fisher & Turner, 1972 [38]	moderate	strong	weak	moderate	weak	weak	weak
Flippin et al., 2021 [35]	weak	moderate	NA	strong	weak	weak	weak
Golos et al., 2011 [36]	moderate	strong	strong	strong	strong	strong	strong
Golos et al., 2013 [37]	weak	weak	weak	moderate	strong	weak	weak
Hendry & Kerr, 1983 [46]	weak	strong	weak	moderate	weak	weak	weak
Iwanaga et al., 2014 [42]	weak	moderate	weak	moderate	moderate	NA	weak
Kirk et al., 2014 [34]	weak	strong	strong	moderate	weak	weak	weak
Kirk & Kirk, 2016 [48]	weak	strong	strong	moderate	weak	strong	weak
Lam et al., 2019 [3]	moderate	strong	strong	moderate	weak	weak	weak
Mische Lawson et al., 2012 [43]	moderate	strong	weak	strong	weak	weak	weak
Moore et al., 1984 [44]	weak	strong	weak	moderate	strong	strong	weak
Mulvey et al., 2018 [30]	strong	strong	weak	moderate	strong	weak	weak
Puder et al., 2011 [45]	strong	strong	strong	strong	moderate	strong	strong
Wang et al., 2020 [32]	weak	strong	strong	moderate	moderate	weak	weak
Zawadzka et al., 2012 [47]	weak	moderate	weak	moderate	weak	weak	weak

Note. Some modifications were made to the EPHPP tool to solve misunderstandings between the raters. *Study design*: Studies that used quasi-experimental design were coded as CCT. *Confounders*: The confounders of interest included age, gender, health status, and pre-intervention score. *Blinding*: In question 2 “Were the study participants aware of the research question?” we chose to code “no” if there was no mention that participants were aware of the research question. This decision was made based on the young age of the participants. *Data collection methods*: The outcome of interest (cognitive or academic measurement) was evaluated. Methods were coded to be “valid” if the validity was mentioned in the article or if there was a citation to a test manual or another article where the validity was reported. Some well-known methods were seen as valid methods without a separate mention (e.g., Wechsler Intelligence Scale for Children or Bayley Scales of Infant and Toddler Development). Methods were coded as “reliable” only if the reliability was measured and reported in that specific data set. *Withdrawals and drop-outs*: In question 1, “Were withdrawals and drop-outs reported in terms of numbers and/or reasons per group?”, if both numbers and reasons were reported it was coded as “yes”, otherwise “no” was selected. Withdrawals and drop-outs were considered as children that did not finish the intervention, i.e., not missing data.

### 3.5. Effect Sizes

Individual effect sizes for each outcome and sub-group within the included studies are reported in Table 2. The effect sizes were presented in four groups based on the assumed severity of the participants learning difficulty:

*Children at-risk for learning difficulties due to family background*. In total, eight of the included studies (one with two separate interventions) [38] investigated the effects of FMS and PA interventions in children with low SES [21,30,34,35,38,44,48], while one was carried out with immigrant children [45]. Two studies assessed cognitive skills as an outcome; one with an FMS only intervention [38] and one with a combined FMS intervention [21]. Both studies demonstrated a beneficial effect of the intervention. The effect was large (*d* = 3.0) for the latter, while an effect size could not be calculated for the former due to the lack of required data. Language skills were assessed in two studies [34,48], both of which demonstrated large beneficial effects of a combined PA intervention (*d* = 0.78–1.57 $\bar{x}$ 1.18). Three of the identified studies included executive functions as an outcome; one demonstrated a small beneficial effect of an FMS only intervention (*d* = 0.48) [30]; one found a significant benefit of a PA only intervention, but an effect size could not be calculated due to the lack of required data [35]; while one did not observe significant effects of an FMS/PA only intervention [45]. Finally, two studies investigated the effects of FMS only interventions on academic skills [38,44]; of which one demonstrated beneficial effects [38]; however, an effect size could not be calculated due to the lack of required data. The null-finding was underpowered to detect a medium effect [44].

*Children with learning difficulties*. In total, three studies assessed the effects of FMS and PA interventions in children at-risk for learning difficulties with low SES backgrounds [36,37,43], two studies on children with learning and perceptual-motor difficulties [39,46], one study on children with delays in language development [49], and one study on children at high risk for ADHD [31]. Three studies assessed cognitive skills as an outcome; two with combined FMS interventions [36,37]; and one with two separate FMS only interventions [39]. Both combined FMS interventions found large beneficial effects on cognitive skills (*d* = 0.78 − 1.87 $\bar{x}$ 1.33); while in two FMS only interventions the effects were assessed on both cognitive and academic skills and no significant effects were found [39]. The null-findings [39] were underpowered to detect a medium effect. Three studies assessed language skills as an outcome [43,46,49]. One study with combined FMS and PA intervention [49] and one study with FMS only intervention [43] reported a beneficial effect on language skills; however, effect sizes could not be calculated due to the lack of required data. The combined FMS intervention observed no significant benefits on language skills [46]. The null-finding [46] was underpowered to detect a medium effect. Finally, one study [31] investigated the effects of an FMS only intervention on executive functions and demonstrated a large beneficial effect (*d* = 1.48).

*Children with learning disabilities.* Three of the included studies investigated the effects of FMS and PA interventions in children with autism spectrum disorder [32,42,47]. One study with two separate interventions involved children with significant delays in cognition, social, motor, speech, or language development [40] and one study was on children with global developmental delay, autism spectrum disorder, or speech development delay [3]. Two studies assessed the effects on cognitive skills with combined FMS interventions [3,47], and both demonstrated beneficial effects; one study with large effects (*d* = 1.15) [47], while the other demonstrated a medium beneficial effect (*d* = 0.52) [3]. Three studies (one with two interventions) [40] assessed the effects on language skills [3,40,42]. A trivial effect was found with the FMS and PA only intervention (*d* = 0.07) [40], whereas the effect was small with the combined FMS and PA intervention (*d* = 0.34) [40] demonstrating significantly greater benefits than the FMS and PA only intervention (*d* = 0.27) [40]. Medium beneficial effects were found with combined FMS intervention (*d* = 0.57) [3]. For an FMS only intervention, while reporting beneficial effects, the effect size could not be calculated [42]. One study assessed the effects of an FMS and PA only intervention on executive functions and demonstrated large beneficial effects (*d* = 1.40) [32]. For academic skills, a trivial beneficial effect was found with the combined FMS and PA intervention (*d* = 0.10) [40] and a small effect with the FMS and PA only intervention (*d* = 0.30) [40]; with no significant differences between the groups.

*Children with physical disabilities.* Two studies assessed the effects of FMS and PA interventions on children with physical disabilities; with one of the studies including children with cerebral palsy and growth hormone deficiency [33]; and one including children who had below average physical development at birth [41]. Both studies assessed the effects on cognitive skills and no significant effects were found, either with combined FMS intervention [33] or with an FMS only intervention [41]. Of these, the former study [33] was underpowered to detect a medium effect.

### 3.6. Methodological Quality and Effect Sizes

Methodological quality and effect sizes are presented in Table 3. Large effects were found in children’s cognitive skills [21,36,47], executive functions [31,32], and language skills [48]. Of these six studies, only one (17%) [36] had a strong methodological quality, while five (83%) [21,31,32,47,48] displayed a weak methodological quality. In addition, only one study that found large effects [32] used outcome measures that were shown to be valid, while three studies [21,36,48] used outcomes that were shown to be reliable. Two of these studies [31,47] used outcome measures that were neither shown to be valid nor reliable. The studies that received a strong rating in terms of data collection methods demonstrated small effects in two studies [30,40], and trivial effects in one study [40]. Five studies reported that the intervention effects were significant [35,38,42,43], or children’s skills improved during the intervention [49]; however, effect sizes could not be calculated due to limited data availability.

**Table 2:** Individual effect sizes for each outcome and subgroup within the included studies.

Reference	Outcome	Sub-Group within Study	Effect Size (*d*)	Sufficient Power to Detect a Medium Effect?
**Children at Risk for Learning Difficulties due to Family Background**				
Draper et al., 2012 [21]	Cognitive skills: Herbst Early Childhood Development Criteria test	Intervention cf. Control	ns.	yes
	Cognitive skills: Herbst Early Childhood Development Criteria test	Within group analysis	3.00	
Fisher & Turner, 1972a; 1972b [38]	Cognitive skills: Slosson Intelligence Test	Intervention (experimental 1 and 2) cf. Control	sign *	no
	Academic skills: Metropolitan Readiness Test	Intervention (experimental 1 and 2) cf. Control	sign *	
Flippin et al., 2021 [35]	Executive functions, sustained attention: on-task behavior	Intervention period cf. Control period	sign. *	yes
Kirk et al., 2014 [34]	Language skills: Pre-school Literacy Individual Growth and Development Indicators, alliteration	Intervention cf. Control	0.78	no
	Language skills: Pre-school Literacy Individual Growth and Development Indicators, picture naming	Intervention cf. Control	0.21	
Kirk & Kirk, 2016 [48]	Lanugage skills: Pre-school Literacy Individual Growth and Development Indicators, alliteration	Intervention cf. Control	0.38	no
	Language skills: Pre-school Literacy Individual Growth and Development Indicators, rhyming	Intervention cf. Control	1.57	
Moore et al., 1984 [44]	Academic skills: The Tests of Basic Experience (TOBE) Level K General Concepts Test	Intervention cf. Control	ns.	no
Mulvey et al., 2018 [30]	Executive functions: Head, Toes, Knees, SKIP -task	Intervention cf. Control	0.48	yes
Puder et al., 2011 [45]	Executive functions, attention: Konzentrations-Hand-lungsverfahren für Vorschulkinder	Intervention cf. Control	ns.	yes
	Executive functions, spatial working memory: subtest taken from the Intelligence and Development Scales	Intervention cf. Control	ns.	
**Children with learning difficulties**				
Berrol, 1984a [39]	Academic skills: Comprehensive Test of Basic Skills	Intervention (Dance/movement therapy) cf. Control	ns.	no
	Executive functions, sustained attention: the Children’s Checking Test (CCT)	Intervention (Dance/movement therapy) cf. Control	ns.	
Berrol, 1984b [39]	Academic skills: Comprehensive Test of Basic Skills	Intervention (Sensory integration activity) cf. Control	ns.	no
	Executive functions, sustained attention: the Children’s Checking Test (CCT)	Intervention (Sensory integration activity) cf. Control	ns.	
Chevalier et al., 2017 [31]	Executive functions, inhibition, Animal Stroop Test	Within group analysis	1.20	no
	Executive functions, inhibition, Animal Stroop Test	Intervention cf. Control	ns.	
	Executive functions, attention: Conners’ Kiddie Continuous Performance Test	Intervention cf. Control	ns.	
		Within group analysis	ns.	
	Executive functions, selective attention: Neuropsychological Assessment Battery (NEPSY).	Intervention cf. Control	1.48	
Coleman & Andersson, 1978 [49]	Language skills: Language recognition inventory	Within group analysis (experimental 1)	improved	n/a
		Within group analysis (experimental 2)	improved	
	Language skills: Assessment of Children’s Language Comprehension	Within group analysis (experimental 1)	improved	
		Within group analysis (experimental 2)	improved	
Golos et al., 2011 [36]	Cognitive skills: Miller Assessment for Preschoolers, complex skills subset	Intervention cf. Control (at-risk or with developmental delays)	1.87	no
	Cognitive skills: Miller Assessment for Preschoolers, non-verbal abilities subset	Intervention cf. Control (at-risk or with developmental delays)	ns.	
Golos et al., 2013 [37]	Cognitive skills: Miller Assessment for Preschoolers, complex skills subset	Within group analysis (2-year group)	ns.	n/a
	Cognitive skills: Miller Assessment for Preschoolers, non-verbal abilities subset	Within group analysis (2-year group)	0.78	
Hendry & Kerr, 1983 [46]	Language skills: grouping of items by shape, size, family name and placing picture cards in a logical story sequence	Intervention cf. Control	ns.	no
	Language skills: recognition of alphabets, short words, geometric shapes, and incomplete pictures	Intervention cf. Control	ns.	
Mische Lawson et al., 2012 [43]	Language skills, grade report: shape recognition	Intervention cf. Control	sign. *	no
	Language skills, grade report: letter recognition	Intervention cf. Control	ns.	
	Language skills, grade report: writing	Intervention cf. Control	ns.	
	Language skills, grade report: color recognition	Intervention cf. Control	ns.	
	Numeracy, grade report: counting	Intervention cf. Control	ns.	
	Numeracy, grade report: number recognition	Intervention cf. Control	ns.	
**Children with learning disabilities**				
Connor-Kuntz & Dummer, 1996a [40]	Academic skills: school readiness composite	Within group analysis (combined, developmentally delayed)	0.1	no
	Langugage skills: Bracken Basic Concept Scale, the direction/position subscale	Within group analysis (combined, developmentally delayed)	0.34	
Connor-Kuntz & Dummer, 1996a; 1996b [40]	Academic skills: school readiness composite	Combined intervention cf. Control	ns.	
	Langugage skills: Bracken Basic Concept Scale, the direction/position subscale	Combined intervention cf. Control (developmentally delayed)	0.27	
Connor-Kuntz & Dummer, 1996b [40]	Academic skills: school readiness composite	Within group analysis (control, developmentally delayed)	0.3	no
	Langugage skills: Bracken Basic Concept Scale, the direction/position subscale	Within group analysis (control, developmentally delayed)	0.07	
Iwanaga et al., 2014 [42]	Language skills: Japanese Miller Assessment for Preschoolers, verbal subset	Within group analysis (individual sensory integration)	ns.	n/a
	Language sklils: Japanese Miller Assessment for Preschoolers, non-verbal subset	Within group analysis (individual sensory integration)	sign. *	
Lam et al., 2019 [3]	Cognitive skills: Cognitive subtest of the Developmental Assessment Chart Revised (DAC-R)	Intervention cf. Control	0.52	yes
	Language skills, verbal comprehension: Reynell Developmental Language Scales (RDLS) Cantonese Version	Intervention cf. Control	0.40	
	Language skills, expressive language: Reynell Developmental Language Scales (RDLS) Cantonese Version	Intervention cf. Control	0.57	
Wang et al., 2020 [32]	Executive functions, working memory: Childhood Executive Functioning Inventory	Intervention cf. Control	0.96	yes
	Executive functions, inhibition: Childhood Executive Functioning Inventory	Intervention cf. Control	1.1	
	Executive functions, regulation: Childhood Executive Functioning Inventory	Intervention cf. Control	1.4	
Zawadzka et al., 2012 [47]	Cognitive skills: Behaviour Observation Scale adapted for children, cognitive subset	Within group analysis	1.15	n/a
**Children with physical disabilities**				
Bala et al., 2013 [41]	Cognitive skills: Raven’s Matrices	Intervention cf. Control (below average development at birth)	ns.	yes
Devesa et al., 2011 [33]	Cognitive skills: The Battelle Developmental Inventory Screening Test, cognitive subset	Within group analysis (pre-treatment period)	ns.	n/a

* sign = significant effects were reported but effect sizes could not be calculated due to limited data availability. improved = beneficial effects were reported with no description of statistical analyses. n/a = not applicable; power analyses could not be conducted for within-group analyses. ns = nonsignifican differences.

**Table 3:** Summary of the relationship between methodological quality and intervention effects.

Intervention	Outcome	Not Significant	Significant, but Effect Sizes Could not be Calculated *	Effect Size (d)			
				Trivial	Small	Medium	Large
FMS	Executive functions	*Berrol, 1984a ^c^ [39];* *Berrol, 1984b ^c^ [39];* *Chevalier et al. 2017 ^c,b^ [31]*			*Mulvey et al. 2018 ^c^ [30]*		*Chevalier et al. 2017 ^b,c^ [31]*
	Language skills	*Mische Lawson et al. 2012 ^c^ [43];* *Iwanga et al. 2014 ^b^ [42]*	*Mische Lawson et al. 2012 ^c^ [43];* *Iwanga et al. 2014 ^b^ [42]*				
	Cognitive skills	*Bala et al. 2013 ^c^ [41]*	*Fisher & Turner, 1972 ^a,c^ [38]*				
	Numeracy	*Mische Lawson et al. 2012 ^c^ [43]*					
	Academic skills	*Moore et al. 1984 ^c^ [44]; Berrol, 1984a ^c^ [39];* *Berrol, 1984b ^c^ [39]*	*Fisher & Turner, 1972 ^a,c^ [38]*				
PA	Executive functions		*Flippin et al. 2021 ^c^ [35]*				
FMS & PA	Executive functions	**Puder et al. 2011 ^c^ [45]**					*Wang et al. 2020 ^c^ [32]*
	Language skills			Connor-Kuntz & Dummer 1996b ^b^ [40]			
	Academic skills				Connor-Kuntz & Dummer 1996b ^b^ [40]		
FMS combined	Language skills	*Hendry & Kerr, 1983 ^c^ [46]*			*Lam et al. 2019 ^c^ [3]*	*Lam et al. 2019 ^c^ [3]*	
	Cognitive skills	*Draper et al. 2012 ^c^ [21];* **Golos et al. 2011 ^c^ [36];***Golos et al. 2013 ^b^ [37]; Devesa et al. 2011 [33] ^b^*				*Golos et al. 2013 ^b^ [37]; Lam et al. 2019 ^c^ [3]*	*Draper et al. 2012 ^b^ [21];* **Golos et al. 2011 ^c^ [36];** *Zawadska et al. 2012 ^b^ [47]*
PA combined	Language skills				*Kirk et al. 2014 ^c^ [34];* *Kirk & Kirk, 2016 ^c^ [48]*	*Kirk et al. 2014 ^c^ [34]*	*Kirk & Kirk, 2016 ^c^ [48]*
FMS & PA combined	Academic skills	Connor-Kuntz & Dummer, 1996a ^d^ [40]		Connor-Kuntz & Dummer 1996a ^b^ [40]			
	Language skills		*Coleman & Andersson, 1978 ^b,e^ [49]*		Connor-Kuntz & Dummer 1996a ^b,d^ [40]		

* Significant effects were reported but effect sizes (*d*) could not be calculated due to limited data availability. ^a^ Two interventions within one study [38] analyzed together. ^b^ Within group analysis. ^c^Intervention compared to control. ^d^ Combined intervention [40] compared to FMS & PA only intervention [40]. ^e^ Beneficial effects were reported with no description of statistical analyses. Methodological quality based on the EPHPP: *weak*, moderate,** strong**.

## 4. Discussion

The present systematic review aimed to investigate the methodological quality and the effects of FMS and PA interventions on cognitive and academic skills in preschool-aged children with SEN. The results demonstrated that only 9% of the included 22 studies had strong methodological quality, while 86% of the studies were rated as methodologically weak. The most often used outcome measures were cognitive and language skills and the largest effect sizes were found for cognitive skills, executive functions, and language skills. The intervention effects appeared to be somewhat dependent on the severity of the difficulty; in cognitive and language skills, the intervention effects were largest in children with minor learning difficulties (i.e., children at-risk due to family background), whereas in executive functions the intervention effects were largest in children with more severe difficulties (i.e., children with learning disabilities). However, due to the vast heterogeneity of the included studies, and rather low methodological quality, it is challenging to summarize the findings in a generalizable manner.

The finding that most of the included studies were methodologically weak is in line with the findings from a previous systematic review that investigated the effects of FMS and PA interventions on cognitive and academic skills in typically developing preschoolers [16]. The low ratings were mostly a result of inadequate reporting practices, especially in participant selection processes, confounders, blinding, data collection methods, and withdrawals [23]. Inadequate reporting practices are a common limitation in other educational interventions as well [50], and, thus, the use of reporting guidelines is highly recommended in the future.

It is recognized that difficulties exist in recruiting adequate sample sizes in children with SEN, and, thus, a large portion of the included studies were underpowered. Nonetheless, the limitations and potential risks of conducting underpowered studies cannot be dismissed (i.e., studies may result in substantially inflated effects or lead to false negative findings) [51]. Thus, results from underpowered studies may lead to erroneous conclusions as per the efficacy of studied interventions, which can subsequently lead to misguided decision making.

When considering the efficacy of PA and/or FMS interventions in children with SEN, the benefits appear to be somewhat dependent on the severity of the difficulty. Indeed, while large improvements in language skills were found for children at-risk due to family background, the effects were trivial-to-medium in children with learning disabilities. Importantly, the intervention effects observed in children at-risk due to family background were comparable to the effects of children without at-risk conditions in the previous review [16]. In cognitive skills, while medium-to-large improvements were demonstrated among all children with SEN, except for children with physical disabilities, a similar trend was observed. Indeed, the effects were progressively smaller in magnitude with increasing severity of the difficulty. It should be noted, however, that the improvements in cognitive skills—regardless of the severity of the difficulty (apart from children with physical disabilities)—were comparable to the ones experienced by typically developing children [16]. These findings indicate that it is easier to support children with more minor difficulties with FMS and/or PA interventions. Indeed, children that are at-risk due to family background, usually lack the opportunities to develop their cognitive [8] and language skills [52] in their home environment, and, thus, they lag behind their average-performing peers. With the right kind of early education support, these children have the possibility to develop their skills, which can have a huge effect on their later success during formal schooling [5,8].

The improvements in executive functions also appeared to be contingent on the severity of the children’s difficulties. In contrast, however, here the effects were large in children with learning difficulties and disabilities, whereas the improvements were small in children at-risk due to family background. In accordance, children with learning difficulties and learning disabilities improved executive functions to a greater extent than typically developing children [16]; which might be reflective of a greater potential to develop executive functions in children with a lower level of executive functions. In line with our findings, studies have demonstrated larger beneficial effects in older children with ADHD in comparison to their typically developing counterparts [53].

Notably, only one of the studies assessed numeracy as an outcome, which was further limited to only a few dimensions of numeracy (i.e., counting and number recognition). Thus, in addition to cognitive and language skills, more studies investigating the effects of FMS and PA interventions on numeracy in children with SEN are required.

In terms of the intervention type, evidence was found for the efficacy of combined interventions for cognitive skills and FMS and/or PA only interventions for executive functions. Due to an insufficient number of studies, comparison between intervention types was possible only for language skills, and in line with our previous findings in typically developing children [16], combined interventions appeared more effective than FMS and PA only interventions. It should be noted, however, that in the combined interventions, the outcome was typically related to the intervention content; thus, these differences might simply stem from the direct practice of the assessed outcome. Finally, the comparison between FMS and PA only interventions could not be done due to the small number of studies and the vast heterogeneity of the participants in the included studies.

### Study Limitations and Strengths

One of the strengths of the present systematic review was that both FMS and PA, as well as combined FMS and PA, interventions were included. In addition, while it is increasingly common for systematic reviews to only include RCTs [54], we included all study designs apart from case studies. This is important, as the use of RCT designs in children with SEN is largely impossible and, thus, remains scarce [55]. Furthermore, the present effect size calculations allowed the quantification and comparison of the intervention effects between the studies. However, some limitations of the present study should be addressed. Namely, only studies that were published in English were included, and some populations were vastly underrepresented, as only two studies assessed children with physical disabilities; making the generalization of the findings unreasonable.

## 5. Conclusions

These results indicate that FMS and PA interventions may be beneficial in the support of cognitive and academic skills in children with SEN. The intervention effects appear to be somewhat dependent on the severity of the difficulty; in cognitive and language skills the intervention effects were largest in children with minor difficulties (i.e., children at-risk due to family background), whereas in executive functions the intervention effects appeared to be largest in children with more severe difficulties (i.e., children with learning disabilities). Moreover, in line with the findings from typically developing children, combined interventions appeared to be more effective compared to FMS and PA only interventions. However, the results should be treated with caution as most of the studies had low methodological quality and displayed vast heterogeneity. More studies including combined interventions as well as FMS and/or PA only interventions in children with SEN are required to confirm the present findings. Finally, adherence to reporting guidelines and the inclusion of a priori power analyses are strongly encouraged for future studies.

## Supplementary Materials

The following supporting information can be downloaded at: https://www.mdpi.com/article/10.3390/brainsci12081001/s1, Table S1: Intervention characteristics of the included studies.
