# Supplementary material for: Cognitive and Academic Outcomes of Fundamental Motor Skill and Physical Activity Interventions Designed for Children with Special Educational Needs: A Systematic Review
Source: Brain Sci. 2022 Jul 28;12(8):1001. doi: 10.3390/brainsci12081001 (PMC9406044; doi:10.3390/brainsci12081001)
Supplement: Supplementary file 1 [file brainsci-12-01001-s001.zip › brainsci-1797567-supplementary.pdf]

Table S1. Intervention characteristics of the included studies

Reference	Study design	Sample size int/con	Reason for SEN	Age (range or mean ± SD), gender (males %)	Cognitive/academic outcome(s) of interest	Intervention exposure	Inter-vention focus	Intervention	Activities in control group
**Children at risk for learning difficulties due to family background**									
Draper et al., 2012 [21], South Africa	CCT	43/40[a]	Economically disadvantaged	Int: age (4.8 ± 0.5 yrs), gender (44.2%)<br>Con: age (4.8 ± 0.6 yrs), gender (67.5%)	Cognitive skills: one subdomain of Herbst Early Childhood Development Criteria test	45-60 min, once a week for eight months	Combined FMS	Little champs program included supervised free play and structured, low-intensity activities that aimed to develop speed, agility, eye-hand/eye-foot coordination and spatial awareness. Children had access to sport equipment, like hula hoops, balls, and bean bags. Different kinds of numeracy and literacy tasks were included in the activities, for example counting balls and collecting items of a particular color. Led by Little Champs coaches.	Business as usual control group.
Fisher & Turner, 1972a [38], USA	CCT	76/26	Culturally disadvantaged and low income	Kindergarten children (Age and gender not reported)	Cognitive skills: Slosson Intelligence Test Academic skills: Metropolitan Readiness Tests, Form A	2 hr, each school day for one academic year	FMS only	The intervention included perceptual-motor training exercises which were derived from the Kephart developmental program. The exercises aimed to develop children's balance, posture, locomotion, laterality, and perceptual-motor coordination. Led by a trained physical education teachers.	Business as usual control group.
Fisher & Turner, 1972b [38], USA	CCT	26/26	Culturally disadvantaged and low income	Kindergarten children (Age and gender not reported)	Cognitive skills: Slosson Intelligence Test<br>Academic skills: Metropolitan Readiness Tests, Form A	2 hr, each school day for half an academic year	FMS only	The intervention included perceptual-motor training exercises which were derived from the Kephart developmental program. The exercises aimed to develop children's balance, posture, locomotion, laterality, and perceptual-motor coordination. Led by a trained physical education teachers.	Business as usual control group.
Flippin et al., 2021 [35], USA	PPD	47	Low sosioeconomic status and/or children with individualised education plan	Age (7.4 ± 0.3 yrs), gender (48.9%)	Executive functions, sustained attention: on-task behaviour measured using Noldus Pocket Observer sofware	Total of three weeks during every school day, in five weeks period	PA only	Classrooms were fitted with variety of kinesthetic equipment (i.e., standing desks, exercise balls, kneel-and-spin desks, under desk pedals, bouncy bands). This equipment incorporated movement in the classroom by enabling various motion activites, such as kneeling, balancing, cycling and standing.	No control group.

Kirk et al., 2014 [34], USA	CCT	51/21	Low socioeconomic status	Int: age (3.8 ± 0.1 yrs), gender (43.1%)<br>Con: age (3.9 ± 0.1 yrs), gender (57.1%)	Language skills: Early Literacy Individual Growth and Development Indicators - Picture Naming, Rhyming and Alliteration	15 min, twice every school day for six months	Combined PA	Intervention combined literacy lessons (e.g., picture naming, rhyming and alliteration) with physical activity aiming to get the children to move in moderate activity for 15 min, twice per day. The physical activity, included movements like marching in place and jumping jacks, and was designed to promote learning. Led by teachers and observed by a member of the research team.	Same content of the literacy lessons, but not integrated with PA.
Kirk & Kirk, 2016 [48], USA	CCT	39/15	Low socioeconomic status	Int: age (4.0 ± 0.2 yrs), gender (35.9%)<br>Con: age (4.1 ± 0.2 yrs), gender (20.0%)	Language skills: Early Literacy Individual Growth and Development Indicators - Picture Naming, Rhyming and Alliteration	30 min, twice every school day for eight months	Combined PA	Intervention combined literacy lessons (e.g., picture naming, rhyming and alliteration) with physical activity aiming to get the children to move in moderate activity for 30 min, twice per day. The physical activity, included movements like marching in place and jumping jacks, and was designed to promote learning. Led by teachers and observed by a member of the research team.	Same content of the literacy lessons, but not integrated with PA.
Moore et al., 1984 [44], USA	CCT	47/38	Low socioeconomic status	Kindergarten children (Age and gender not reported)	Academic skills: The Tests of Basic Experience Level K General Concepts Test	30 min, every school day for 12.5 weeks	FMS only	The intervention followed Capon's Perceptual-motor Lesson Plans. In each lesson there was three different stations with a different perceptual-motor task, and the children circulated the stations in order. Led by trained aides and teachers.	Children had 30 min undirected PA at the playground
Mulvey et al., 2018 [30], USA	RCT	50/57	Low income	Age (5.14 ± 0.81 yrs), gender (45.8%)	Executive functions: Head Toes Knees Shoulders	30 min, twice a week for six weeks	FMS only	Successful Kinesthetic Instruction for Preschoolers (SKIP) intervention, that aimed to improve fundamental motor skills, including both locomotor and object control skills through cognitively demanding physical activities. The program considered children's individual needs by within-task variation and diverse instruction and feed-back.	Business as usual control group.
Puder et al., 2011 [45], Switzerland	RCT	342/310	High immigrant population	Int: age (5.2 ± 0.6 yrs), gender (51.2%)<br>Con: age (5.2 ± 0.6 yrs), gender (48.7%)	Executive functions, attention: Konzentrations-Hand-lungsverfahren für Vorschulkinder Spatial working memory: Intelligence and Development Scales	45 min, four times a week for one academic year	FMS and PA only	The intervention included physical acitivity sessions, about nutrition, sleep and media use. In addition, several home activities and extracurricular physical activity were offered for the children. The physical activity lessons were primarily held in the classroom or in the gym, and they aimed to increase coordination and endurance in a playful manner. Equipment, like balls and skipping ropes were also used. Mainly led by teachers, who were supported by a trained health promoter.	Business as usual control group.

**Children with learning difficulties**									
Berrol, 1984a [39], USA	CCT	21/24	Learning and perceptual-motor problems and low sosioeconomic status	Int: age (6.5 yrs), gender (57.1%)<br>Con: age (6.7 yrs), gender (54.2%)	Executive functions, sustained attention: Children's Checking Test<br>Academic skills: Comprehensive Test of Basic Skills	30 min, three times a week for 13 weeks	FMS only	Dance/Movement Therapy (DMT): The intervention aimed to develop sensory-perceptual-motor function through dance and movement. Fundamental motor skills, balance, posture, object control, visual form perception, and sensory discrimination were trained while reducing hyperactivity. Various materials and equipment were used, e.g., rhythm instruments, bean bags and geometric forms. Led by a researcher and an assistant.	Business as usual control group.
Berrol, 1984b [39], USA	CCT	23/24	Learning and perceptual-motor problems and low sosioeconomic status	Int: age (6.5 yrs), gender (52.2%)<br>Con: age (6.7 yrs), gender (54.2%)	Executive functions, sustained attention: Children's Checking Test<br>Academic skills: Comprehensive Test of Basic Skills	30 min, three times a week for 13 weeks	FMS only	Sensory Motor Activities (SMA): Intervention included sensory integration activities (tactile, vestibular, proprioceptor). Activities (e.g., posture and balancing training, obstacle courses, form and space perception) were aimed to elicit automatic reactions in contrast to planned responses. Children circulated activity stations individually spending 5 minutes at each station. Led by teachers and supervised by an occupational therapist.	Business as usual control group.
Chevalier et al., 2017 [31], Canada	CCT	7/8	High risk of ADHD	Int: age (5.7 ± 0.3 yrs), gender (42.9%)<br>Con: age (5.9 ± 0.3 yrs), gender (100%)	Executive functions, inhibition: Animal Stroop test<br>Selective attention: The Developmental Neuropsychological Assessment Battery<br>Attention: Conners' Kiddie Continuous Performance Test	Three times a week for 12 weeks	FMS only	The motor-cognitive remediation program (MCRP) included kinesthetics, visual and auditory sensory modalities and its aim was to develop attention control through movement. The program was divided into six groups of main activities and consisted of 30 different sensimotor activities. Led by teachers and parents.	Business as usual control group.

Coleman & Andersson, 1978 [49], USA	PPD	Group 1: n = 9 Group 2: n = 7	Developmentally delayed and language-impaired children	Group 1: 5.1 yrs Group 2: 4.6 yrs	Language skills: Assessment of Children's Language Comprehension, and language recognition inventory	2.5 hr, four days a week for eight weeks	Combined FMS and PA	The language tasks, coverinng five language areas (nouns, verbs, adjectives, prepositions, syntax), were structured so that they elicited motor behaviors and whole-body movements (i.e., "show me a dog running", or "make the chair fly"). Led by speech-language pathologists.	No control group.
Golos et al., 2011 [36], Israel	CCT	27/54	Children at-risk for developmental delays and with low sosioeconomic status	Int: age (4.3 ± 0.3 yrs), gender (100%) Con: age (4.3 ± 0.3 yrs), gender (100%)	Cognitive skills: Miller Assessment for Preschoolers complex skills and non-verbal abilities subsets	One monitoring group: Once a week for 30 min for 10 to 12 sessions during 8 month	Combined FMS	Children were assigned to one or more monitoring groups, which were: manual dexterity, fundamental motor skills, graphomotor skills, and cognitive skills. Ball and balance skills were practiced in the motor skill group. The monitoring groups were led by trained developmental aide.	Business as usual control group. (Students have only few oppor-tunies for play and PA)
Golos et al., 2013 [37], Israel	PPD	28[b]	Children at-risk for developmental delays and with low sosioeconomic status	Preschoolers: age (4.2 ± 0.3 yrs), gender (100%) Kindergarteners: age (5.0 ± 0.3 yrs), gender (100%)	Cognitive skills: Miller Assessment for Preschoolers complex skills and non-verbal abilities subsets	One monitorin group: Once a week for 30 min for 10 to 12 sessions during 8 month	Combined FMS	Children were assigned to one or more monitoring groups, which were: manual dexterity, fundamental motor skills, graphomotor skills, and cognitive skills. Ball and balance skills were practiced in the motor skill group. The monitoring groups were led by trained developmental aide.	No control group.
Hendry & Kerr, 1983 [46], Canada	CCT	8/8	Learning disabled children	Kindergarten children (Age and gender not reported)	Language skills: Grouping of items by shape, size, family name and placing picture cards in a logical story sequence. Recognition of alphabets, short words, geometric shapes, and incomplete pictures	1-1.5 hr, once a week for six months	Combined FMS	The intervention combined motor and cognitive training, as the children practiced fundamental movements and created own stories through these movements. The story was then written in simple sentences visible for the children, and they were asked to associate the words to the correct movements and answer questions about the story. Led by one of the authors.	Business as usual control group.

Mische Lawson et al., 2012 [43], USA	CCT	18/15	Children at high risk due to difficulties with speech, behavior management or other concerns, and with low sosioeconomic status	Age (3-5 yrs), gender (60.6%)	Language skills: Grade reports from shape, letter and color recognition, and writing Numeracy: Grade reports from counting and number recognition	10 min, four days a week for six weeks	FMS only	The intervention sessions included a 10-minute modified form of yoga "Yoga Reflex Integration" -video immediately followed by some motor activity (games, fine motor activities etc.). The video motivated children to do different animal poses, that develop reflex integration and yoga postures. Two teachers, an occupational therapist and paraprofessional were trained to led the intervention.	Control croup participated to half-day curriculum
**Children with learning disabilities**									
Connor-Kuntz & Dummer, 1996a [40], USA	RCT	11.11	Children with cognitive and language delays	Int: age (4.8 ± 0.8 yrs), gender (46.2%) Con: age (4.9 ± 0.6 yrs), gender (81.8%)	Academic skills: school readiness composite Language skills: Bracken Basic Concept Scale, the direction/position subscale	30 min, three times a week for eight weeks	Combined FMS and PA	Physical activities included body management, fundamentla motor skills, games, and dance. Activities were combined with language and numeracy training. The motor activities were enriched with learning of concepts like quantity, comparisons, direction, letters, numerals, and colors. Led by researchers.	Active control group (i.e., Connor-Kuntz et al., 1996b)
Connor-Kuntz & Dummer, 1996b [40], USA	RCT	11.11	Children with cognitive and language delays	Int: age (4.9 ± 0.6 yrs), gender (81.8%) Con: age (4.8 ± 0.8 yrs), gender (46.2%)	Academic skills: school readiness composite Language skills: Bracken Basic Concept Scale, the direction/position subscale	30 min, three times a week for eight weeks	FMS and PA only	Physical activities included body management, fundamentla motor skills, games, and dance. Led by researchers.	Active control group (i.e., Connor-Kuntz et al., 1996a)
Iwanaga et al., 2014 [42], Japan	CCT	8/12	Children with high-functioning autism spectrum disorder (Autistic, asperger's, or pervasive developmental disorder)	Int: age (4.7 ± 0.8 yrs), gender (100%) Con: age (4.7 ± 0.6 yrs), gender (83.3%)	Language skills : The Japanese version of the Miller Assessment for Preschoolers, verbal and non-verbal subsets	60 min, once a week for 9.3 ± 1.0 months	FMS only	During sensory integrative therapy (SIT), a therapist and one child acted in a room with equipment like a swing, balance beam, ladder, trampoline, and a ball pit. This equipment and activities presented different sensory and kinetic opportunities and challenged child's postural and bilateral motor control. The specific activities were individually planned for the children. Led by a researcher (certified SI therapist and an occupational therapist) individually.	No control group.

Lam et al., 2019 [3], China	CCT	60/60	Children diagnosed with SEN (i.e., children with global developmental delay, autism spectrum disorder, speech developmental delay, or others, such as ADHD)	Int: age (4.08 ± 0.55 yrs), gender (78.3%) Con: age (4.05 ± 0.67 yrs), gender (76.7%)	Cognitive skills: Cognitive subtest of the Developmental Assessment Chart Revised Language skills, verbal comprehension: Reynell Developmental Language Scales Expressive language: Cantonese (Hong Kong) version, respectively	1-2 hr, each session for one academic year (center-based training). Every school day for one academic year (school-based training).	Combined FMS	Children received an Individual Education Plan (IEP) which was implemented in school environment. Children also received center-based therapeutic training services: All children reveiced 21-22 sessions of speech therapy, and 33-34 sessions of cognitive and social skills training. Additionally, 30 children received 21-22 sessions of fine motor skills training, and 30 children received 20–21 sessions of gross motor skills training (14 children received both fine motor and gross motor training). The school-based IEP was led by teachers who were trained by a multidisciplinary team of professionals. The center-based sessions were led by speech therapists, a special childcare worker, occupational therapists, and physiotherapists.	Business as usual control group.
Wang et al., 2020 [32], China	CCT	18/15	Autism spectrum disorder	Int: age (5.11 ± 0.65 yrs), gender (83.3%) Con: age (4.70 ± 0.70 yrs), gender (86.7%)	Executive functions: Childhood Executive Functioning Inventory	40 min, five days a week for 12 weeks	FMS and PA only	Each session included a warm-up, basketball skill learning, basketball games and a cool-down, resulting a total of 30 min of moderate-intensity physical activity per session. Led by certified physical educators.	Business as usual control group.
Zawadzka et al., 2012 [47], Poland	PPD	20	Autistic children	Age (4.8 yrs; range 3 to 6 yrs), gender (85%)	Cognitive skills: Behaviour Observation Scale, adapted for children, cognitive subset	90 min, once a week for six months	Combined FMS	The intervention combined occupational therapy with Veronica Sherborne Developmental Movement Therapy, which is a treatment program for autistic children. Intervention included play, games and excercises that developed cognitive, emotional, social and motor domains of psychomotor development. Led by therapists.	No control group.
**Children with physical disabilities**									
Bala et al., 2013 [41], Serbia	PPD	63/62[c]	Weight and height below average at birth	Age (6.13 ± 1.04 yrs), gender (63.6%), not reported for each group	Cognitive skills: Raven Progressive Matrices assessment test	60 min, twice a week for one academic year	FMS only	Kinesiological activities program, including e.g. calisthenics, posture control, exercises for motor abilities, sports games, dance and yoga.	Business as usual control group.

Devesa et al., 2011 [33], Spain	PPD	11[d]	Cerebral palsy and growth hormone defiency	Age (4.12. ± 1.31 yrs), gender (63.6%)	Cognitive skills: the Battelle Developmental Inventory Screening Test, cognitive subset	45 min, five days a week for four months	Combined FMS	Psychomotor and cognitive stimulation that were adapted to the specific needs of each child. Psychomotor stimulation involved tasks aimed at improving e.g., tonic–postural control, laterality, static and dynamic balance, motor coordination, spatial and temporal orientation, and gross motor skills. Cognitive stimulation aimed to improve e.g., attention, perception, memory and concept learning.	No control group.

CCT = Controlled clinical trial

PPD = Pre-post design

Studies that have active control goup was used as within group design in current analysis.

[a] Article included two different studies. Only study 2 (with cognitive function) is discussed here.

[b] Only children in the longer group included

[c] Only children that were below average are used in this analysis

[d] Only pretreatment period is used in this analysis
